# Fine Mapping, Transcriptome Analysis, and Marker Development for *Y_2_*, the Gene That Conditions β-Carotene Accumulation in Carrot (*Daucus carota* L.)

**DOI:** 10.1534/g3.117.043067

**Published:** 2017-06-29

**Authors:** Shelby Ellison, Douglas Senalik, Hamed Bostan, Massimo Iorizzo, Philipp Simon

**Affiliations:** * Department of Horticulture, University of Wisconsin–Madison, Wisconsin 53706; † Vegetable Crops Research Unit, United States Department of Agriculture-Agricultural Research Service, Madison, Wisconsin 53706; ‡ Plants for Human Health Institute, Department of Horticultural Science, North Carolina State University, Kannapolis, North Carolina 28081

**Keywords:** carotenoids, *Daucus carota* L., genotyping-by-sequencing, RNA-sequencing, QTL

## Abstract

Domesticated carrots, *Daucus carota* subsp. *sativus*, are the richest source of β-carotene in the US diet, which, when consumed, is converted into vitamin A, an essential component of eye health and immunity. The *Y_2_* locus plays a significant role in beta-carotene accumulation in carrot roots, but a candidate gene has not been identified. To advance our understanding of this locus, the genetic basis of β-carotene accumulation was explored by utilizing an advanced mapping population, transcriptome analysis, and nucleotide diversity in diverse carrot accessions with varying levels of β-carotene. A single large effect Quantitative Trait Locus (QTL) on the distal arm of chromosome 7 overlapped with the previously identified β-carotene accumulation QTL, *Y_2_*. Fine mapping efforts reduced the genomic region of interest to 650 kb including 72 genes. Transcriptome analysis within this fine mapped region identified four genes differentially expressed at two developmental time points, and 13 genes differentially expressed at one time point. These differentially expressed genes included transcription factors and genes involved in light signaling and carotenoid flux, including a member of the *Di19* gene family involved in Arabidopsis photomorphogenesis, and a homolog of the *bHLH36* transcription factor involved in maize carotenoid metabolism. Analysis of nucleotide diversity in 25 resequenced carrot accessions revealed a drastic decrease in diversity of this fine-mapped region in orange cultivated accessions as compared to white and yellow cultivated and to white wild samples. The results presented in this study provide a foundation to identify and characterize the gene underlying β-carotene accumulation in carrot.

Carotenoids are a subgroup of isoprenoid compounds produced by algae, bacteria, fungi, and plants that absorb light during photosynthetic light capture. They also provide photoprotection, attract pollinators and seed dispersers, and serve as precursors for the production of important downstream compounds including norisoprenoids, apocarotenoids, strigolactones, and abscisic acid (Walter and Strack 2011; Alder *et al.* 2012; Ruiz-Sola and Rodríguez-Concepción 2012; Ellison 2016). Humans, and most animals, cannot produce carotenoids and therefore they must acquire them through their diet (Walter and Strack 2011). Colorful carotenoids accumulated by some animals also ensure reproductive success during courtship or may be involved in defense mechanisms (Cazzonelli 2011). Humans convert provitamin A carotenoids, such as α- and β-carotene, into vitamin A, which is critical for maintaining normal vision, a healthy immune system, and effective cellular communication and differentiation (Fraser and Bramley 2004; Biesalski *et al.* 2007; Rao and Rao 2007).

Carotenoids in plants are synthesized in differentiated plastids including the chloroplasts of green tissues and chromoplasts of flower petals, fruits, and roots (Yuan *et al.* 2015). The storage roots of orange carrots, *Daucus carota* L. (2*n* = 2x = 18), accumulate high concentrations of α- and β-carotene, making carrot one of the richest sources of dietary provitamin A carotenoids. Indeed, orange carrots account for 28% of the β-carotene and 67% of α-carotene, derived from plant sources, in the US diet (Simon *et al.* 2009; Just *et al.* 2009). However, the genetic mechanisms that control substantial carotene accumulation in carrot, particularly β-carotene, are only beginning to be understood.

In carrot, the *Y* and *Y_2_* loci explain most of the phenotypic variation among white, yellow, and orange storage roots (Laferriere and Gabelman 1968; Buishand and Gabelman 1979; Simon 1996; Bradeen *et al.* 1997; Just *et al.* 2007; Iorizzo *et al.* 2016). In this model, *Y_Y_2__* conditions white, *yyY_2_*_ yellow, *Y_ y_2_y_2_* pale orange, and *yyy_2_y_2_* orange roots (Figure 1). Previous research identified several Quantitative Trait Loci (QTL) associated with carotenoid accumulation, and mapped the *Y* and *Y_2_* loci to chromosomes 5 and 7, respectively (Santos and Simon 2002; Just *et al.* 2009; Cavagnaro *et al.* 2011), and a SCAR marker was developed for *Y_2_* to facilitate marker-assisted selection for beta-carotene (Bradeen *et al.* 1997; Bradeen and Simon 1998), which can be challenging to visually phenotype in certain segregating populations and in diverse genetically uncharacterized diverse germplasm, especially in early development. Recently, researchers utilized Genotyping-by-Sequencing (GBS) and RNA-sequencing to identify a candidate gene for the *Y* locus, DCAR_032551 (Iorizzo *et al.* 2016). Interestingly, this candidate is not a carotenoid biosynthetic gene, but rather shares homology with the Arabidopsis homolog *PEL* (*Pseudo-Etiolation in Light*), which is involved in the regulation of photomorphogenesis and de-etiolation (Ichikawa *et al.* 2006). Several carrot studies have found associations between carotenoid content and carotenoid biosynthetic genes, including a study by Arango *et al.* (2014), which identified a *Carotene Hydroxylase* (*CYP97A3*) homolog that contributed to increased carotenoid content due to increased amounts of α-carotene. Further, a candidate gene association analysis by Jourdan *et al.* (2015) suggested total carotenoid and β-carotene quantities were significantly associated with the genes *Zeaxanthin Epoxidase* (*ZEP*), *Phytoene Desaturase* (*PDS*), and *Carotenoid Isomerase* (*CRTISO*). To verify whether these genes underlie the *Y_2_* locus, a whole-genome integrative approach was used.

**Figure 1:**
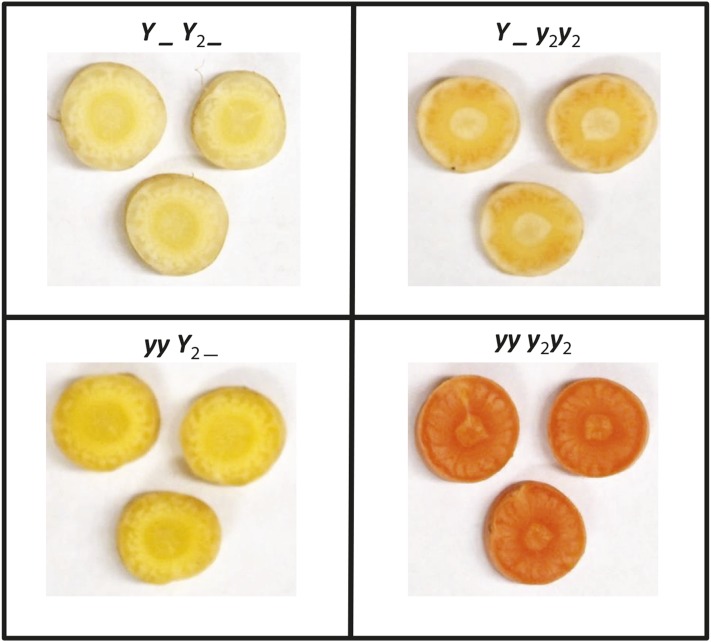
Visual appearance of the four phenotypic classes of carrot storage root color conferred by the *Y* and *Y_2_* loci. *Y_Y_2__* (white) top left, *yyY_2__* (yellow) bottom left, *Y_y_2_y_2_* (pale orange) top right, and *yyy_2_y_2_* (dark orange) bottom right.

A better understanding of β-carotene accumulation and the genetic architecture of the *Y_2_* locus will contribute to the genetic improvement of nutritional content in carrots and may provide novel targets to pursue increased carotenoid accumulation in other crop species. To address this objective, we utilized the recently published carrot genome to identify candidate genes and explore the genetic control of beta-carotene accumulation in a mapping population segregating for the *Y_2_* locus. While the *Y_2_* gene accounts for most of the accumulation of both alpha- and beta-carotene, in orange carrots, we focused on β-carotene accumulation in this study since five additional QTL were found to account for α-carotene accumulation in a mapping study (Santos and Simon 2002), with *Carotene Hydroxylase* having a particularly large effect (Arango *et al.* 2014). Additionally, we used RNA-sequencing to identify differentially expressed genes within the *Y_2_* fine-mapped region as well as in the 2-*C*-methyl-d-erythritol 4-phosphate (MEP) and carotenoid pathways. Further, we evaluated SNPs from white, yellow, and orange resequenced carrot accessions to determine if nucleotide diversity was reduced around the *Y_2_* locus among orange carrots. Finally, we developed codominant markers to assist in selection for beta-carotene accumulation in segregating populations.

## Materials and Methods

### Plant materials

The F_4_ population 74,146 was derived from a cross between USDA carrot inbred line B493 (Simon and Peterson 1990), an orange-rooted line, and QAL (Queen Anne’s Lace), a wild-type white-rooted carrot from the United States. Plants were grown the summer of 2013 at the University of Wisconsin, Hancock Agricultural Research Station, and 213 roots were selected for phenotyping and genotyping. Population 74,146 was preliminarily evaluated and found to be homozygous recessive *yy*, but segregating for root color associated with the *Y_2_* locus. An additional 192 samples from the 74,146 population were grown at the University of Wisconsin, Walnut Street Greenhouse and used for fine mapping. To analyze the segregation ratios between parents and progeny, two F_4:5_ populations (98,024 and 98,026) derived from self-pollination of the 74,146 population were grown in the summer of 2013 at the UW Madison Hancock Research Station, and an additional two similarly derived populations (98,029 and 98,032) were grown during the winter of 2014–2015 at the University of California, Desert Research and Extension Center.

### Carotenoid and color evaluation

Carotenoid content was quantified using lyophilized root tissue for HPLC analysis as modified from Simon and Wolff (1987) and Simon *et al.* (1989). Briefly, 0.1 g of lyophilized carrot root tissue was crushed and then soaked in 2.0 ml of petroleum ether at 4°. After 15 hr, 300 µl of the petroleum ether extract was added to 700 μl of methanol, eluted through a Rainin Microsorb-MV column, and analyzed on a Millipore Waters 712 WISP HPLC system. Synthetic β-carotene (Sigma-Aldrich, St. Louis, MO) was used in each independent run as a reference standard for calibration. β-Carotene was quantified by absorbance at 450 nm. Concentrations are described in microgram per gram dry weight (DW). Additionally, phenotypic estimates of carotenoid content were taken using a visual categorical scale. Carrot roots were cross cut at mid-root and then categorized into two phenotypic groups: yellow or orange. Goodness-of-fit for a single gene model was calculated using visual categories.

### GBS

Total genomic DNA of individual plants was isolated from lyophilized leaves of 4-wk old plants following the protocol described by Murray and Thompson (1980) with modifications by Boiteux *et al.* (1999). DNA was quantified using Quantus PicoGreen ds DNA Kit (Life Technologies, Grand Island, NY), and normalized to 10 ng/μl. GBS, as described by Elshire *et al.* (2011), was carried out at the University of Wisconsin–Madison Biotechnology Center with minimal modification and half-sized reactions. Briefly, DNA samples were digested with ApeKI, barcoded, and pooled for sequencing, and 80–85 pooled samples were run per single Illumina HiSequation 2000 lane, using paired-end, 100 nt reads and v3 SBS reagents (Illumina, San Diego, CA). Paired-end sequencing reads were preprocessed with bb.tassel (https://github.com/dsenalik/bb) to add barcodes to the reverse reads for TASSEL compatibility. The TASSEL-GBS pipeline version 4.3.7 was used to call SNPs as described by Bradbury *et al.* (2007) and Glaubitz *et al.* (2014). SNPs were filtered for <10% missing data for genotype and marker, >10% minor allele frequency, and no more than two alleles. This set of 78,850 SNPs was submitted to dbSNP at NCBI under BioProject PRJNA348698. Any remaining missing genotype calls were imputed using Beagle v4.0 with parameters burnin-its = 10, phase-its = 10, and impute-its = 10 (Browning and Browning 2016). Imputed markers were further filtered for minimum allele frequency >0.3 and maximum allele frequency <0.7, leaving 33,712 SNPs. Marker-trait associations were carried out with molecular markers considered as fixed effects in a linear model implemented in the GLM function of TASSEL (Bradbury *et al.* 2007). The carrot genome assembly v2.0 (GenBank accession LNRQ01000000) was used as a reference to identify marker locations (Iorizzo *et al.* 2016). The genome-wide significance threshold was determined by the Bonferroni method, *P* ≤ 0.05 (Bland and Altman 1995).

### Linkage map construction

Heterozygous SNPs, with an allele ratio expected to be 1:1, were eliminated if the ratio of the two alleles was <0.3 or >0.7, leaving 2999 high quality markers for linkage mapping (Supplemental Material, Table S1 in File S1). Genetic linkage analysis and map construction was executed in JoinMap 4 (Van Ooijen 2006) as previously described (Cavagnaro *et al.* 2014). The 74,146 map was analyzed as an F_2_ population. Markers ascertained to be the result of false double recombination events were identified using CheckMatrix version 248 (http://www.atgc.org/XLinkage) and removed. The following parameters were used for the calculation: Haldane’s mapping function, LOD ≥3.0, REC frequency ≤0.4, goodness-of-fit jump threshold for removal of loci = 5.0, number of added loci after which a ripple was performed = 1, and third round = no. At LOD >10, with <10% missing data for marker and genotype, 616 markers were grouped into nine linkage groups (Figure S1)

### QTL mapping

QTL analysis was carried out using the R package R/qtl (Broman and Sen 2009). For the single QTL model interval analyses, genotype probabilities were calculated with a step value of 1 over the entire linkage map. The “scanone” function used the normal phenotype model (model = “normal”) and the Haley-Knott regression method (method = “hk”) as parameters. After running 1000 permutations with an assumed genotyping error rate of 0.001, a LOD of 4.01 was set as the QTL significance threshold. Confidence intervals for each QTL were defined as the 1.5 LOD drop off flanking the peak of the QTL. Linkage maps and QTL were drawn using Mapchart 2.1 software (Voorrips 2002).

### Fine mapping

Based on visual inspection of recombination events depicted in the TASSEL viewer, and confidence intervals identified in the QTL analysis, fine-mapping was conducted with an additional 192 individuals using 13 newly developed SNPs spanning positions 32,973,430–34,339,369 on chromosome 7. A set of 13 primer pairs were designed using Primer3 (Untergasser *et al.* 2012), targeting specific loci spanning the genomic region associated with β-carotene accumulation. Marker and primer coordinates have been adjusted to reflect the most recent genome release (*D. carota* v2.0, GenBank accession LNRQ01000000). DNA was extracted from freeze-dried leaves as previously described. PCR and Sanger sequencing were performed as described in Iorizzo *et al.* (2012). Primer information can be found in Table S2 in File S1.

### Nucleotide diversity

An analysis of seven wild (white; *Y_Y_2__*), seven cultivated nonorange (white; *Y_Y_2__* or yellow; *yyY_2__*), and 11 cultivated orange (*yyy_2_y_2_*) resequenced carrot accessions (Table S3 in File S1, Iorizzo *et al.* 2016) identified 1,378,264 SNPs on Chromosome 7 that were used to estimate nucleotide diversity (*π*) in TASSEL (Bradbury *et al.* 2007) with the method described by Nei and Li (1979). Nucleotide diversity was calculated using a sliding window = 1000 and 500 SNPs per step. Genome coordinates were adjusted to reflect the most recent carrot genome (*D. carota* Ver.2, Bioproject PRJNA268187).

### Transcriptome analysis

Carrot root tissue was collected from three yellow (*yyY_2_Y_2_*) and three orange (*yyy_2_y_2_*) pigmented biological replicates, plants from the progenitor F_2_ population of population 74,146, at 40 (time point one) and 80 (time point two) days after planting (DAP). Two time points were sampled to detect potential variation in expression across development. Time point one corresponds to the onset of visual detection of carotenoid accumulation in the storage root, and time point two corresponds to the onset of the plateau in carotenoid accumulation. Total RNA was extracted from storage root tissue using the TRIzol Plus RNA Purification Kit (Life Technologies, Carlsbad, CA) in accordance with the manufacturer’s protocol. Contaminating DNA was removed with the TurboDNA-free kit (Life Technologies, Carlsbad, CA). RNA quantity and integrity was confirmed with an Experion RNA StdSens Analysis kit (Bio-Rad, Hercules, CA). All samples had RQI values >8.0.

For each sample, a 133-nt insert size paired-end library was prepared at the Biotechnology Center, UW-Madison. Libraries were sequenced on Illumina HiSeq2000 lanes using 2 × 100 nt reads. Reads were filtered with Trimmomatic version 0.32 with adapter trimming and using a sliding window of length ≥50 and quality ≥28, *i.e.*, “ILLUMINACLIP:adapterfna:2:40:15 LEADING:28 TRAILING:28 MINLEN:50 SLIDINGWINDOW:10:28.”

Short reads from each replicate were independently mapped against the carrot genome sequence (GenBank accession LNRQ01000000.1) using Bowtie2 (Langmead and Salzberg 2012) and Tophat2 (Kim *et al.* 2013) (Table S4 in File S1). Reads for each gene (exon level) available from the V1.0 gene annotation of the carrot genome (Iorizzo *et al.* 2016) were quantified with the featurecounts (Liao *et al.* 2014) standalone package, using only reads that mapped uniquely to the genome.

Pearson correlations between samples were calculated between technical replicates (Table S5 in File S1) and samples A11, A11r, B3, B3r, B4, B4r, C3, C5, C6, and E1 were discarded due to high correlation with noncorresponding replicates (Table S6 in File S1). Differentially expressed genes (DEGs) were identified in four unique comparisons: (1) yellow time point one *vs.* orange time point one, (2) yellow time point two *vs.* orange time point two, (3) orange time point one *vs.* orange time point two, and (4) yellow time point one *vs.* yellow time point two, using a Negative Binomial test implemented in the DESeq package (Anders and Huber 2010).

### Candidate gene sequence alignment

Four primer pairs spanning the candidate gene DCAR_026175 were developed with Primer3 (Table S2 in File S1, Untergasser *et al.* 2012). Extracted DNA from a yellow (*Y_2_Y_2_*) and orange (*y_2_y_2_*) plant from the 71,746 population was amplified using DCAR_026175-specific primers to produce high-quality PCR amplicons and purified using BigDye Terminator v3.1 Cycle Sequencing Kit (Life Technologies, Carlsbad, CA) as per the manufacturer’s instructions. DNA sequencing of the PCR amplicons was performed at the University of Wisconsin Biotechnology Center. Sequences were aligned using Sequencher version 5.0 DNA sequence analysis software (Gene Codes Corporation, Ann Arbor, MI).

### Cleaved amplified polymorphic sequences (CAPS) marker development

PCR amplicons containing SNPs were identified during fine-mapping and sequence data were used to locate possible restriction enzyme site polymorphisms using the program NEBcutter V2.0 (Vincze *et al.* 2003). Two polymorphic PCR amplicons, 4135 and 4144, that differed for restriction enzyme sites *Apo*I and *Ape*KI, were used to develop CAPs markers. For CAPs marker 4135*^Apo^*^I^, cleavage of the amplified fragment was carried out according to manufacturer’s recommendations. In summary, the digestion took place at 37° for 15 min using the following conditions: 15 μl of the PCR product, 2 μl (1×) buffer CutSmart (New England Biolabs), and 1 μl (5 U) of the restriction enzyme *Apo*l (New England Biolabs) in a final volume of 20 μl. For CAPs marker 4144c*^Ape^*^KI^, cleavage was carried out at 75° for 15 min using the following conditions: 15 μl of the PCR product, 2 μl (1×) buffer 3.1 (New England Biolabs), and 1 μl of (5 U) of the restriction enzyme *Ape*KI (New England Biolabs) in a final volume of 20 μl. Digestion products were separated on a 2% agarose gel in 1× TAE buffer.

### Data availability

SNPs from the 74,146 mapping population were deposited in dbSNP under BioProject PRJNA348698. Raw reads from the 29 carrot transcriptomes were deposited under BioProject PRJNA350691. Resequenced carrot accessions were obtained from Bioproject PRJNA291976. The authors state that all other data and necessary code for confirming the conclusions presented in the article are available as Supplemental Material.

## Results

### Phenotypic evaluation and inheritance

Segregation ratios for the F_4_ 74,146 population and F_4:5_ families fit a single gene model with yellow color dominant to orange (Table 1 and Table S7 in File S1). These results agree with previous studies indicating that the dominant *Y_2_* allele reduces β-carotene accumulation resulting in yellow root color (Buishand and Gabelman 1979; Simon 1996; Just *et al.* 2009). β-Carotene content for 158 yellow and 52 orange roots averaged 0.5 and 77.7 μg g^−1^ DW, respectively (Table S7 in File S1).

**Table 1:** Color segregation ratios observed in carrot populations derived from family 74,146 that segregates for the *Y_2_* gene

Population	Location	Parent Root Color	Inferred Parental Genotype	Yellow	Orange	Expected Segregation Ratio	*χ*^2^	*P*-Value
74,146	WI 2012	Yellow	*Y_2_y_2_*	158	52	3:1	0.006	0.94
98,024	WI 2013	Yellow	*Y_2_y_2_*	79	17	3:1	2.72	0.1
98,026	WI 2013	Orange	*y_2_y_2_*	0	32	0:1	0	1
98,029	CA 2014	Yellow	*Y_2_Y_2_*	102	0	1:0	0	1
98,032	CA 2014	Orange	*y_2_y_2_*	0	134	0:1	0	1

### Mapping and QTL analysis

The v4.0 TASSEL GBS pipeline analysis of population 74,146 called 512,427 SNPs. Filtering and imputation left 33,712 high quality SNPs scored in 210 plants. The distribution of markers across the nine chromosomes ranged from 2029 to 5168, with an average of one GBS marker every 11.3 kb (Table S8 in File S1).

**Figure 2:**
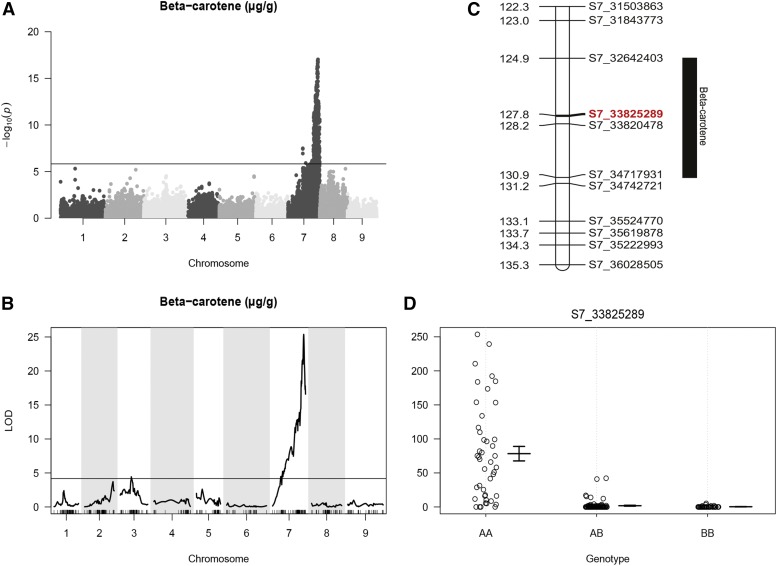
Mapping and QTL analysis for carrot β-carotene content. (A) Manhattan plot for β-carotene concentration. Horizontal line indicates Bonferroni correction for multiple testing. (B) QTL analysis for β-carotene concentration. Horizontal line indicates QTL significance threshold. (C) Linkage map of the distal end of chromosome 7 showing the QTL for β-carotene content. Black bar covers the 1.5 LOD drop off region. The most significant marker, S7_33,825,289, is highlighted in red. (D) Marker effect plot for S7_33,825,289, within the QTL region on chromosome 7. AA represents the homozygous recessive genotype (*y2y2*), while AB and BB represent the heterozygous and homozygous dominant genotypes (*Y_2__*), respectively. Vertical axis indicates the quantity of beta-carotene (μg/g).

To identify the genetic region that includes the *Y_2_* trait locus, HPLC data (β-carotene content) was used to identify marker-trait associations with the 33,712 GBS SNPs. Genome-wide tests to identify significant association were carried out using a standard GLM analysis in TASSEL (Elshire *et al.* 2011). Inspection of the Q-Q plot confirmed no inflation in *P*-values (data not shown). A region of high significance was found on the distal end of chromosome 7 for β-carotene content, as observed by Just *et al.* (2009) (Figure 2A and Table S9 in File S1).

QTL analysis was also carried out for β-carotene concentration. After filtering for missing data and segregation distortion, 616 high quality SNPs were called in 176 plants. The distribution of markers across the nine linkage groups ranged from 33 to 118 (Table S10 in File S1), with an average of one marker every 1.9 cM (Figure S1 and Table S11 in File S1).

A single QTL on the distal arm of chromosome 7 was identified for β-carotene concentration, with a maximum LOD value of 25.4 with the nearest marker at S7_33,825,289 (Figure 2, B and C). The QTL for beta-carotene concentration explained 48.5% of the phenotypic variation. This QTL overlaps with the region identified by GLM analysis. An effect plot for the most significant marker, S7_33,825,289, was used to determine the contribution of allelic states (A, H, B) on the phenotypic expression of the trait (Figure 2D). In the homozygous recessive state (AA) this marker was associated with an increase of >80 μg g^−1^ DW β-carotene.

### Fine mapping

**Figure 3:**
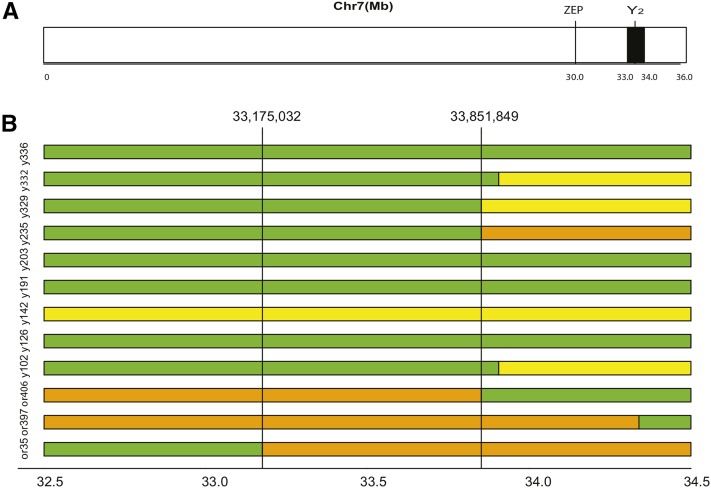
Fine-mapping the *Y_2_* locus on chromosome 7. (A) Chromosome 7 including the location of *Y_2_*, delimited by SNP markers S7_33,019,341 and S7_33,979,543 in black, and the nearest carotenoid biosynthetic gene, *ZEP* at 30,085,761. (B) Fine-mapping of the *Y_2_* region, delimited by SNP markers S7_33,175,032 and S7_33,851,849. Orange homozygous recessive, yellow heterozygous, and yellow homozygous dominant genotypes are represented by orange, green, and yellow bars, respectively. Genotypes used for fine mapping are shown to the left of the colored bars. Horizontal axis values indicate physical location (megabase).

Flanking the region of highest significance on chromosome 7, two recombinants were found to border a region of ∼1.0 Mb (Figure 3A). The flanking markers of this region were S7_33,019,341 and S7_33,979,543. The locations of recombination events were used as starting points for fine mapping.

The inclusion of 192 additional plants for fine mapping reduced the candidate region from 1 Mb to ∼650 kb on chromosome 7 between markers 33,175,032 and 33,851,849 (Figure 3B). Samples with linkage blocks between markers 33,175,032 and 33,851,849 harboring the “B” and “H” alleles had low β-carotene levels and were classified as Yellow, Y, whereas samples associated with the “A” allele had high β-carotene content and were classified as Orange, Or (Table S7 in File S1). These results are consistent with the hypothesis that high β-carotene is controlled by the *Y_2_* locus (Just *et al.* 2009). This region is included within the previously mapped QTL region associated with the *Y_2_* trait (Cavagnaro *et al.* 2011; Just *et al.* 2009).

In total, 72 genes have been predicted in the 650 kb fine-mapped region (Iorizzo *et al.* 2016; Table 2 and Tables S12 and S13 in File S1). Only one gene from the MEP or carotenoid pathway, *1-Deoxy-D-xylulose 5-phosphate reductoisomerase*, (*DXR*), was found in the region of interest. The most highly represented genes in the region of interest were related to nucleotide or DNA binding, while other common groups included biosynthetic processes, transporter and kinase activities.

**Table 2:** The 72 genes within the *Y_2_* fine-mapped region. Subject description is the top Blastp hit in SwissProt or TrEMBL

Gene Annotation	Subject Description	Gene Annotation	Subject Description
DCAR_026103*^a^*	Butyrate–CoA ligase AAE11	DCAR_026139	Pantothenate kinase 2
DCAR_026104	Ceramide kinase	DCAR_026140	Iron-sulfur cluster assembly protein 1
DCAR_026105	Pyruvate decarboxylase 2	DCAR_026141	Probable boron transporter 2
DCAR_026106	30S ribosomal protein S4	DCAR_026142	GDSL esterase/lipase
DCAR_026107*^a^*	Auxin response factor 6	DCAR_026143	Phosphoenolpyruvate/phosphate translocator 1
DCAR_026108*^b^*	Replication protein A 70 kD DNA-binding subunit C	DCAR_026144	Glutathione S-transferase 3
DCAR_026109*^b^*	Probable galactinol-sucrose galactosyltransferase 5	DCAR_026145	Protein EXECUTER 2
DCAR_026110	Chalcone synthase	DCAR_026146	Replication protein A 70 kD DNA-binding subunit E
DCAR_026111*^a^*	Chalcone synthase 4	DCAR_026147	Replication protein A 70 kD DNA-binding subunit A
DCAR_026112	40S ribosomal protein SA	DCAR_026148*^b^*	Nucleolar GTP-binding protein 1
DCAR_026113	Calcium-dependent protein kinase 19	DCAR_026149	Secretory carrier-associated membrane protein
DCAR_026114	Probable alpha-mannosidase I MNS5	DCAR_026150	Probable calcium-binding protein CML16
DCAR_026115*^a^*	Calmodulin-binding protein 60 A	DCAR_026151	Wall-associated receptor kinase 3
DCAR_026116*^a^*	Calmodulin-binding protein 60 B	DCAR_026152	NEDD8-specific protease 1
DCAR_026117	LIM domain-containing protein WLIM1	DCAR_026153	Glutaredoxin-C1
DCAR_026118	ABC transporter C family member 1	DCAR_026154*^a^*	Homeobox protein knotted-1-like 6
DCAR_026119	Nucleobase-ascorbate transporter 6	DCAR_026155	Serine/threonine protein phosphatase 2A
DCAR_026120	Probable lysine-specific demethylase ELF6	DCAR_026156	Factor of DNA methylation 3
DCAR_026121	Rac-like GTP-binding protein 3	DCAR_026157	Protein MICRORCHIDIA 6
DCAR_026122	Probable cyclic nucleotide-gated ion channel 20	DCAR_026158	Pantothenate kinase 2
DCAR_026123	Putative FBD-associated F-box protein At5g56400	DCAR_026159	Probable apyrase 7
DCAR_026124	Protein HAPLESS 2-B	DCAR_026160	Short-chain dehydrogenase TIC 32
DCAR_026125	U-box domain-containing protein 19	DCAR_026161	FKBP12-interacting protein of 37 kD
DCAR_026126*^a^*	Transcription factor bHLH36	DCAR_026162	Tryptophan aminotransferase-related protein 2
DCAR_026127*^a^*	Probable polygalacturonase	DCAR_026163	Oleosin 5
DCAR_026128	T-complex protein 1 subunit gamma	DCAR_026164	Protein LATERAL ORGAN BOUNDARIES
DCAR_026129	Peptidyl-prolyl *cis*-trans isomerase	DCAR_026165	Putative pentatricopeptide repeat-containing protein
DCAR_026130	Protein EXECUTER 2, chloroplastic	DCAR_026166	Histone deacetylase 6
DCAR_026131	Serine/threonine-protein kinase HT1	DCAR_026167	Histone deacetylase 6
DCAR_026132	Peroxisomal membrane protein PEX14	DCAR_026168	Histone deacetylase 6
DCAR_026133	1-deoxy-D-xylulose 5-phosphate reductoisomerase	DCAR_026169*^a^*	U-box domain-containing protein 12
DCAR_026134*^a^*	Rhodanese-like domain-containing protein 10	DCAR_026170*^a^*	Primary amine oxidase
DCAR_026135*^a^*	Probable inactive poly [ADP-ribose] polymerase	DCAR_026171	Ferric reduction oxidase 8
DCAR_026136	Serine/threonine-protein kinase ATM	DCAR_026172	Protein NBR1 homolog
DCAR_026137	Dof zinc finger protein DOF5.6	DCAR_026173*^a^*	Uncharacterized protein
DCAR_026138	50S ribosomal protein L12	DCAR_026174	Oxygen-evolving enhancer protein 3
		DCAR_026175*^b^*	Protein DEHYDRATION-INDUCED 19 homolog 5

a Genes differentially expressed at one time point

b Genes differentially expressed at both time points

### Nucleotide diversity

Resequencing data were used to evaluate nucleotide diversity between wild white (*Y_Y_2__*), white (*Y_Y_2__*), and yellow (*yyY_2__*) cultivated, and orange (*yyy_2_y_2_*) cultivated accessions in the region associated with high β-carotene accumulation on chromosome 7. Several chromosomal regions had reduced nucleotide diversity, comparing cultivated and wild accessions. However only one region, encompassing the *Y_2_* fine mapped region was associated with a decrease in diversity, comparing orange cultivated with nonorange (yellow and white) cultivated carrots (Figure S2).

### Transcriptome analysis

β-Carotene content ranged from 202 to 846 μg g^−1^ DW among the three orange (*y_2_y_2_*) biological replicates (plants), whereas it ranged from 4 to 30 μg g^−1^ DW among the three yellow (*Y_2_Y_2_*) biological replicates (Table S14 in File S1). Transcriptome analysis comparing orange and yellow samples at both time points detected 3626 differentially expressed genes (DEGs) (Tables S15 and S16 in File S1). Within the 650 kb fine-mapped region containing the *Y_2_* gene, 13 DEGs were identified at one time point (Table 2 and Tables S17 and S18 in File S1) while only four genes were differentially expressed at both time points—*Replication protein A 70 kD DNA-binding subunit C* (DCAR_026108), *Galactinol-sucrose galactosyltransferase 5* (DCAR_026109), *Nucleolar GTP-binding protein 1* (DCAR_026148), and *Protein DEHYDRATION-INDUCED 19 homolog 5* (*Di19*) (DCAR_026175) (Table 2 and Tables S19 and S20 in File S1).

To date, 68 gene families involved in the MEP or carotenoid biosynthetic pathway have been identified in carrot (Iorizzo *et al.* 2016). Within the MEP and carotenoid pathways six genes were differentially expressed at one time point (*PSY-1*, *CYP707a-2*, *NSY-2*, *CYP707a-1*, *PSY-3*, and *CCD1-1*), and two were differentially expressed at both time points (*GPPS-1* and *LUT5*), where both genes were downregulated in orange carrots relative to yellow (Figure S3 and Table S18 in File S1). The only MEP or carotenoid gene within the *Y_2_* fine mapped region, *DXR*, was not differentially expressed. Between time point one and time point two, 10 genes were differentially expressed in orange plants, and only one gene was differentially expressed between yellow plants (Table S18 in File S1).

### DCAR_026175 sequence analysis

**Figure 4:**
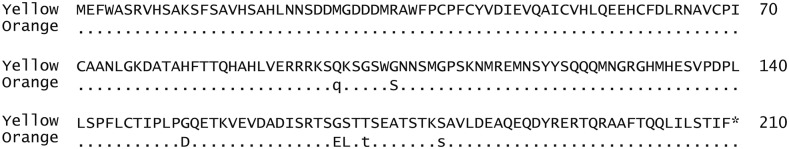
Comparison of the amino acid sequences of DCAR_026175 between yellow and orange carrot roots in population 74,146. The full length amino acid sequence is shown with dots indicating no change, lowercase letters represent synonymous mutations, and uppercase letters denote nonsynonymous mutations.

Of the differentially expressed genes in the *Y_2_* fine mapped region, only DCAR_026175 was differentially expressed at both time points and had lowered expression in orange genotypes, consistent with the recessive nature of the orange phenotype. Sequence analysis comparing a yellow homozygous plant with an orange homozygous plant identified three synonymous mutations, and four nonsynonymous mutations in the protein coding region of the candidate gene DCAR_026175 (Figure 4).

### CAPs marker development

**Figure 5:**
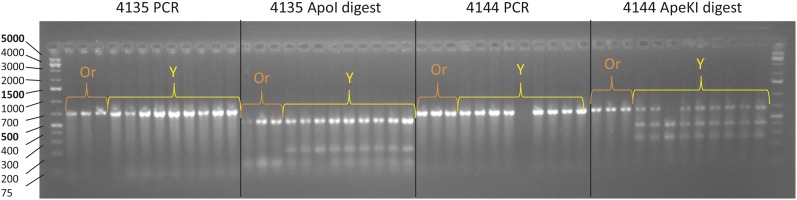
Gel electrophoresis image of the two CAPs markers within the *Y_2_* fine-mapped region. Amplicon 4135 is within DCAR_026133 and amplicon 4144 is within DCAR_026127. GeneRuler 1 kb Plus DNA ladder sizes are shown to the left of the gel image. Or indicates orange samples, and Y indicates yellow samples from population 74,146.

Several genes within the 650 kb fine mapped region were analyzed for sequence polymorphisms to develop CAPs markers that can be used to aid in marker-assisted selection for β-carotene accumulation. DCAR_026127 and DCAR_026133 had SNPs within restriction enzyme sites for *Ape*KI and *Apo*I, respectively, and these polymorphisms were used to develop CAPs markers (Table S2 in File S1). CAPs markers cosegregated with color for all samples used to fine-map the *Y_2_* region, and with all domesticated samples in a panel of unrelated yellow and orange carrot accessions (Figure 5, Figure S4, and Table S21 in File S1).

## Discussion

β-Carotene accumulation has been extensively studied in many crop and model species including Arabidopsis (Ruiz-Sola and Rodríguez-Concepción 2012), maize (Owens *et al.* 2014), and tomato (Yuan *et al.* 2015). However, in carrot, which is one of the highest naturally occurring sources of β-carotene, the genetic regulation of accumulation is still unclear.

Previous studies in carrot have mapped QTL for carotenoid accumulation with AFLP markers (Santos and Simon 2002), and/or have utilized candidate genes from the MEP and carotenoid biosynthetic pathways to identify the genetic control of β-carotene accumulation (Just *et al.* 2007, 2009; Jourdan *et al.* 2015; Iorizzo *et al.* 2016). However, this is the first study to use a whole-genome approach along with a transcriptome analysis to better understand the regulation of high β-carotene accumulation. Analysis of an F_4_ mapping population segregating for β-carotene content found a single highly significant region on the distal region of chromosome 7 associated with an 80-fold increase in β-carotene, agreeing with previous studies (Just *et al.* 2009; Cavagnaro *et al.* 2011). This region accounts for 48.5% of the phenotypic variation in β-carotene content within the 74,146 population. Remaining phenotypic variation may be explained by smaller effect QTL modifying β-carotene accumulation (Santos and Simon 2002) and by nongenetic sources. Utilizing 192 additional samples, the region of interest was fine mapped to a region of 650 kb. Analysis of the nucleotide diversity in 25 resequenced white, yellow and orange carrots revealed a drastic decrease in nucleotide diversity surrounding the fine mapped *Y_2_* region in orange carrots, compared to white and yellow, indicating directional selection for the *Y_2_* haplotype in domesticated orange carrots. Within this region, 72 genes have been annotated and only one gene, *DXR*, is part of the isoprenoid pathway. A previous study by Jourdan *et al.* (2015) found total carotenoid and beta-carotene content were significantly associated with polymorphisms within the genes *ZEP* (DCAR_025735), *PDS* (DCAR_016085), and *CRTISO* (DCAR_013459); however, none of these genes are located within the *Y_2_* region that was fine mapped in our study. It is likely that the limited density of sequences/markers, 17 genes distributed across the whole genome, used by Jourdan *et al.* (2015), combined with the fact that *ZEP*, which is located ∼3 Mb upstream from the *Y_2_* region of interest, may have resulted in a false association, due to linkage to the selective sweep that likely took place around the *Y_2_* region that we characterized in this study.

Although *DXR* mapped to the region of interest in our study, it was not differentially expressed at either time point. *DXR* has been shown to be important in carotenoid flux regulation. In *Arabidopsis*, it appears to be a rate-determining enzyme and overexpression in seedlings increases carotenoid production (Estévez *et al.* 2001; Carretero-Paulet *et al.* 2006). However, a study in tomato did not find evidence for a limiting role of *DXR* in carotenoid biosynthesis (Rodríguez-Concepción *et al.* 2001). Similarly, a recent study in carrot found that *DXR* has a limited regulatory role on carotenoid accumulation in carrot roots and leaves (Simpson *et al.* 2016). We therefore believe *DXR* is not the underlying candidate gene for β-carotene accumulation within the *Y_2_* region.

### Differential expression in the Y_2_ fine-mapped region

Differential expression was analyzed at two time points in an analysis of yellow (*yyY_2_Y_2_*) and orange (*yyy_2_y_2_*) storage root tissue. Within the *Y_2_* fine-mapped region, 17 genes were differentially expressed, and, of these, only four were differentially expressed at both time points, *Replication protein A 70 kDa DNA-binding subunit C* (DCAR_026108), *Galactinol-sucrose galactosyltransferase 5* (DCAR_026109), *Nucleolar GTP-binding protein 1* (DCAR_026148), and *Protein DEHYDRATION-INDUCED 19 homolog 5* (*Di19*) (DCAR_026175) (Table 2). Of these four genes, only *Di19* was downregulated in orange (*y_2_y_2_*) storage roots, as would be expected for a recessive trait. The combination of fine-mapping with transcriptome analysis points to *Di19* as a strong candidate for the *Y_2_* gene.

Sequence analysis of the DCAR_026175 coding region identified three synonymous and four nonsynonymous SNPs between homozygous yellow and orange carrot roots (Figure 4). The nonsynonymous mutations occur in the C-terminal domain of *Di19*, and represent candidate mutations altering expression and downstream function of the gene. Members of the *Arabidopsis* *Di19* gene family can function in an ABA-independent fashion and are regulated by other abiotic stimuli such as member *AtDi19-7*, which has been implicated in regulating light signaling and responses (Milla *et al.* 2006). Consequently, altered expression of *Di19* could potentially influence the coordinated production of chlorophyll and carotenoids that occurs during photomorphogenesis. Of the remaining 13 genes in the region of interest that were differentially expressed at one time point, one interesting candidate, DCAR_026126, shares homology with a *bHLH36* transcription factor. Ye *et al.* (2015) observed that *bHLH* expression was highly correlated with carotenoid metabolism, suggesting a complex underlying regulatory network controls carotenoid flux. Similarly, Endo *et al.* (2016) found *bHLH1* from citrus has a similar function to *Arabidopsis* activation-tagged *bri1 suppressor 1* (*ATBS1*) interacting factor (*AIF*), which may be directly involved in carotenoid metabolism in mature citrus fruit. Based on these observations, future studies should examine the functional role of DCAR_026175 and DCAR_026126 in β-carotene regulation and accumulation.

### Differential expression in the isoprenoid pathway

Within previously characterized isoprenoid genes, but outside of the *Y_2_* region, five genes (*PSY-1*, *CYP707a-2*, *NSY-2*, *CYP707a-1*, and *PSY-3*) were differentially expressed at time point one, and one gene at time point two (*CCD1-1*) (Table S18 in File S1). *PSY* is considered a rate-limiting enzyme in carotenoid biosynthesis and changes in expression have been linked to flux in the pathway (Maass *et al.* 2009; Rodríguez-Vilalon *et al.* 2009). Plants typically have several *PSY* genes that exhibit tissue-specific expression such as in tomato and citrus where *PSY-1* is found in fruits, *PSY-2* in leaves, and *PSY-3* in roots (Peng *et al.* 2013; Fantini *et al.* 2013). We found *PSY-1* to be downregulated and *PSY-3* to be upregulated in orange carrot storage roots as compared to yellow roots. Other studies in carrot have found a relationship between increased *PSY-1* and *PSY-2* expression between white and nonwhite carrots (yellow or orange), but this relationship begins to dissolve when comparing expression between yellow and orange roots (Bowman *et al.* 2014; Wang *et al.* 2014). Therefore, expression of *PSY-1* and *PSY-2* in root tissue may be more associated with total carotenoid content, including xanthophylls and carotenes, rather than β-carotene specifically. It is also likely that *PSY-1* and *PSY-2* have less of a role in carotenoid accumulation in the roots than in other anatomical parts including leaves and fruits.

Two genes in this study, both outside of the *Y_2_* region, were differentially expressed at both time points (*LUT5* and *GPPS-1*). A study by Arango *et al.* (2014) concluded an 8-nt insertion in the *LUT5* gene in orange carrots contributed to dysfunction of this gene, and, consequently, increased carotenoid content due to α-carotene accumulation. Our study found *LUT5* expression was not detectable in orange carrots, which agrees with Arango *et al.* (2014) and provides further evidence for the role of *LUT5* in carotene accumulation. Interestingly, *LUT5* (DCAR_023843) is on chromosome 7 at position 6,061,642 Mb, which is located near the second lowest region of nucleotide diversity, in orange compared to nonorange (white and yellow) carrots (Figure S2). Since a decrease in expression of *LUT5* leads to an increase in α-carotene and total carotenes, it is likely this region of the genome also underwent selection to increase total carotenoid content in carrot storage roots. Therefore both *Y_2_* and *LUT5* have played an important role in the accumulation of carotenoids in carrot. Within the *Y_2_* fine mapped region, 10 genes were differentially expressed between the two time points in orange roots and one gene in yellow roots (Table S18 in File S1), illustrating the importance of understanding carotenogenesis across development and root maturity to fully appreciate the complexity of carotenoid accumulation. Future studies including coexpression network analysis should be conducted across multiple time points during plant growth to better understand carotenoid accumulation throughout storage root development.

### Narrowing down potential Y_2_ candidates

In many plants, carotenoid biosynthetic genes are responsible for the accumulation of carotenoids; however, there are several other mechanisms outside of this pathway that regulate accumulation. These mechanisms include transcriptional regulation of carotenoid biosynthetic and degradation genes, regulation of sequestration and storage, plastid biogenesis, and regulatory genes (Giuliano and Diretto 2007; Li and Yuan 2013; Yuan *et al.* 2015; Nisar *et al.* 2015). Several genes including *DDB1* and *CHCR* in tomato and *Or* in cauliflower are not carotenoid biosynthetic genes, but rather they regulate or sequester carotenoids, resulting in accumulation (Lieberman *et al.* 2004; Lu *et al.* 2006; Kilambi *et al.* 2013). Similarly, a recent analysis of 98 plastidal methylerythritol phosphate (MEP) and carotenoid pathway genes in carrot revealed no overlap with the *Y* or *Y_2_* QTL (Iorizzo *et al.* 2016). Instead, the *Y* candidate gene, DCAR_032551, is involved in the regulation of photomorphogenesis and de-etiolation. Within the fine mapped region of *Y_2_* there are several transcription factors, including DCAR_026126, DCAR_026130, DCAR_026137, and DCAR_026145, that could potentially be involved in the transcriptional regulation of β-carotene accumulation. These candidate genes are also worthy of further investigation in future studies. Further, it is important to note that differential expression of the candidate gene for *Y_2_* may not have been detected in our analysis. Potential reasons for this are: (1) the developmental time point or tissue type to capture differential expression may not have been evaluated, (2) protein levels of translated *Y_2_* mRNA may not correlate with mRNA expression levels, as has been reported in other studies (Payne 2015), and (3) the number of transcriptome biological replications was too small to detect important but subtle differences in gene expression.

Beyond expanding the number of root developmental stages and number of biological replicates evaluated, an alternative tactic to further narrow the list of *Y_2_* candidates includes taking an association mapping approach which may drastically narrow the region of interest. Initial estimates of LD in carrot (unreported) show rapid decay (1–2 kb) making it an ideal crop for association mapping given the correct marker density. Another potential strategy to narrow candidates is to analyze the corresponding steady state levels of candidate proteins since this may be more accurate of gene expression than mRNA transcript abundance. Well-supported candidates should then be subject to functional assays such as complementation studies or genome editing to validate their function in β-carotene accumulation. Additionally, improved carrot genome annotation may strengthen or reduce support for candidates identified by increasing the depth of coverage in this region, and sequencing the transcriptomes of various pigmented carrots at different developmental stages and in different tissue types may lead to novel annotations that were not identified in the initial annotation efforts.

### Marker development for β-carotene accumulation

Previous research identified a QTL for β-carotene and total carotenoids on a ∼30 cM region on chromosome 7. Visual phenotyping of β-carotene accumulation due to *Y_2_* segregation is challenging in certain segregating carrot populations and at early developmental time points, so within this QTL a codominant marker Y2mark was created to facilitate marker-assisted breeding (Bradeen *et al.* 1997; Bradeen and Simon 1998). Y2mark maps to the carrot genome at position 35,382,784 Mb, ∼2 Mb away from the newly fine-mapped *Y_2_* region. We have developed two closely linked codominant markers, 4135^Apol1^ and 4144^ApeKI^, to more accurately select *y_2_y_2_* plants with increased β-carotene accumulation. These markers have been tested not only within the mapping population, but also in a group of unrelated genetic materials, and have proven to be very accurate in predicting orange and nonorange phenotypes (Figure S4).

By enhancing our knowledge of the regulation of biosynthetic processes and flux through the carotenoid pathway, undoubtedly new possibilities will emerge to utilize this information to accelerate plant improvement. Special interest in carotenoid biosynthesis in plants is attributed to the highly beneficial chemical properties of carotenoids compounds that are well recognized in promoting human health, for example, their antioxidant properties and provitamin A activity. Our research has utilized an integrative, whole-genome approach to better understand β-carotene accumulation in carrot, while looking beyond known biosynthetic genes to discover novel mechanisms regulating carotenoid biosynthesis, accumulation and storage.

### Conclusions

In this study, we report the first fine mapping of a major locus, *Y_2_*, controlling β-carotene accumulation in carrot. This strategy reduced the previously described region from ∼30 cM, based upon QTL analysis, with ∼1 recombination event every 388 kb (Iorizzo *et al.* 2016) to 650 kb. In the fine-mapped region, we identified 17 differentially expressed genes, of which only four were differentially expressed at both time points. Genes within the *Y_2_* fine-mapped region, and especially those with differential expression, are of particular interest for candidate gene identification and functional analyses in the future. Additionally, the marker development for the *Y_2_* region provides a convenient molecular tool to discriminate low and high β-carotene content carrots.

## Supplementary Material

Supplemental material is available online at www.g3journal.org/lookup/suppl/doi:10.1534/g3.117.043067/-/DC1.
